# 伴有*EGFR*基因敏感突变的难治性肺肠型腺癌1例

**DOI:** 10.3779/j.issn.1009-3419.2025.102.44

**Published:** 2025-11-20

**Authors:** WANG Xinyi, MU Ning, LI Feng’e, LIU Mei, XU Yue, WU Shengnan, LV Huan, MA Chunhua

**Affiliations:** 300121 天津，天津市人民医院（南开大学第一附属医院）肿瘤5科; Department of Oncology 5, Tianjin Union Medical Center (The First Affiliated Hospital of Nankai University), Tianjin 300121, China

**Keywords:** 肺肿瘤, *EGFR*突变, 原发耐药, TROP2, 免疫治疗, Lung neoplasms, *EGFR* mutation, Primary resistance, TROP2, Immunotherapy

## Abstract

肺肠型腺癌（pulmonary enteric adenocarcinoma, PEAC）是非小细胞肺癌（non-small cell lung cancer, NSCLC）的一种特殊亚型，其组织形态学及免疫表型与转移性结直肠腺癌高度相似，发病机制及标准治疗策略尚未明确。本文报道1例伴表皮生长因子受体（epidermal growth factor receptor, *EGFR*）外显子19缺失突变且程序性细胞死亡配体1（programmed cell death ligand 1, PD-L1）高表达的PEAC患者，先后接受第一代（埃克替尼）、第二代（阿法替尼）及第三代（阿美替尼）EGFR-酪氨酸激酶抑制剂（EGFR-tyrosine kinase inhibitors, EGFR-TKIs）治疗均未见明显疗效，滋养层细胞表面抗原2-抗体偶联药物（trophoblast cell surface antigen 2-antibody-drug conjugate, TROP2-ADC）、免疫检查点抑制剂（immune checkpoint inhibitors, ICIs）联合贝伐珠单抗治疗在本例中亦疗效有限。我们基于该病例的临床特征及治疗反应，结合已发表的文献综述了PEAC的病理特征、基因突变谱和治疗现状，重点探讨*EGFR*突变型PEAC的治疗困境和研究前景，以期为未来临床实践和相关研究提供参考。

## Abstract (en)

Pulmonary enteric adenocarcinoma (PEAC) is a distinct subtype of non-small cell lung cancer (NSCLC) whose histomorphology and immunophenotype closely resemble those of metastatic colorectal adenocarcinoma; its pathogenesis and standard treatment strategies have yet to be clearly established. Here, we reported a case of PEAC harboring epidermal growth factor receptor (*EGFR*) exon 19 deletion mutation with high programmed cell death ligand 1 (PD-L1) expression. The patient showed no meaningful response to EGFR-tyrosine kinase inhibitors (EGFR-TKIs), including the first-generation (Icotinib), the second-generation (Afatinib) and the third-generation (Aumolertinib). Trophoblast cell surface antigen 2-antibody-drug conjugate (TROP2-ADC) and immune checkpoint inhibitors (ICIs) combined with Bevacizumab also resulted in limited efficacy. Based on the clinical features and treatment response of this case, we reviewed the published literature about the pathological characteristics, mutational landscape, and current therapeutic approaches for PEAC, with a particular focus on the therapeutic challenges and future research directions for *EGFR*-mutant PEAC, aiming to provide insights for clinical practice and further studies.

肺肠型腺癌（pulmonary enteric adenocarcinoma, PEAC）是具有独特病理特征的非小细胞肺癌（non-small cell lung cancer, NSCLC）的一种特殊亚型，在原发性肺腺癌中约占0.68%^[1]^。2015年世界卫生组织（World Health Organization, WHO）将PEAC定义为肿瘤组织中超过50%的肿瘤细胞呈肠道样形态且临床排除消化道肿瘤转移，同时至少表达一种肠道分化相关的免疫组织化学标志物，如尾型同源盒转录因子2（caudal type homeobox transcription factor 2, CDX2）、细胞角蛋白20（cytokeratin 20, CK20）或黏蛋白2（mucin 2, MUC2）等^[2]^。PEAC在分子特征方面也与常规肺腺癌存在显著差异。与常规肺腺癌相比，Kirsten大鼠肉瘤病毒癌基因同源物（Kirsten rat sarcoma viral oncogene homolog, *KRAS*）和人类表皮生长因子受体2（human epidermal growth factor receptor 2, *HER2*）突变以及错配修复（mismatch repair, MMR）蛋白功能缺陷在PEAC中更为常见，而表皮生长因子受体（epidermal growth factor receptor, *EGFR*）突变的发生率则相对较低^[3]^。目前，PEAC患者预后尚不明确，但有研究^[1]^提示其可能具有更强的侵袭性，且临床发病时间晚于常规肺腺癌。由于缺乏大样本的临床研究，PEAC的发病机制和标准治疗方案尚不清晰，临床治疗多参照NSCLC的治疗原则，根据不同的临床分期选择以手术切除和化疗为主的综合治疗模式^[4]^。本研究报道了1例*EGFR*基因外显子19缺失突变伴程序性细胞死亡配体1（programmed cell death ligand 1, PD-L1）高表达的PEAC患者，经EGFR-酪氨酸激酶抑制剂（EGFR-tyrosine kinase inhibitors, EGFR-TKIs）、滋养层细胞表面抗原2-抗体偶联药物（trophoblast cell surface antigen 2-antibody-drug conjugate, TROP2-ADC）及免疫治疗等多线治疗后，病情仍在短期内持续快速进展。我们基于该病例的临床特征及治疗反应，结合已发表的文献综述了PEAC的病理特征、基因突变谱和治疗现状，重点探讨*EGFR*突变型PEAC的治疗困境和研究前景，以期为未来临床实践和相关研究提供参考。

## 1 病例资料

患者女，78岁，2025年4月7日因“胸闷、憋气”就诊于天津市人民医院。患者因“颈椎疼痛伴上肢麻木”于2024年10月首次就诊于当地医院，计算机断层扫描（computed tomography, CT）平扫示左肺上叶占位性病变，考虑恶性可能。2024年11月转诊至天津市肿瘤医院，胸部增强CT示左肺上叶占位伴纵隔淋巴结、颈椎及胸椎转移。为进一步明确诊断，于支气管镜下获取左肺上叶尖段肿瘤组织标本，免疫组化结果提示：甲状腺转录因子-1（thyroid transcription factor-1, TTF-1）（+），Napsin A（+），P40（-），CD56（-），病理诊断为肺腺癌。基因检测提示*EGFR*基因外显子19缺失突变（c.2236_2250del, p.E746_A750del）。临床诊断为伴有*EGFR*敏感突变的晚期肺腺癌（cT3N2M1，IV期）。2024年11月26日起予第一代EGFR-TKIs埃克替尼，每次125 mg，每日3次。1个月后因病情控制不理想，剂量增至每次250 mg，每日3次。2025年2月初患者自觉胸闷憋气，呼吸困难，外院胸部CT示新发双侧大量胸腔积液，疗效评估为疾病进展。基因检测复检（血液）提示*EGFR*外显子19缺失突变（c.2236_2250del, p.E746_A750del），未见耐药相关突变。2025年2月6日起更换第二代EGFR-TKIs阿法替尼，每次40 mg，每日1次。患者胸闷、憋气症状缓解不明显，为积极治疗就诊于天津市人民医院。患者既往无吸烟、饮酒史，否认高血压、糖尿病、冠心病及慢性肺部疾病等基础疾病病史，否认家族遗传病史、酗酒史或精神疾病史。2025年4月7日复查胸部增强CT示左肺上叶及右肺下叶实变影，双肺、双侧胸膜及纵隔多发转移瘤，双肺癌性淋巴管炎，双侧胸腔积液（图1A、1B）。胸水送检基因检测及病理检查。基因检测结果提示*EGFR*外显子19缺失突变（c.2236_2250del, p.E746_A750del），PD-L1蛋白表达水平（CST EIL3N抗体）肿瘤细胞阳性比例分数（tumor proportion score, TPS）为80%，综合阳性分数（combined positive score, CPS）为82。病理检查（胸腔积液）提示腺癌，结合免疫组化符合PEAC（图2）。免疫组化： CK-pan（+）， CK5/6（散在间皮+，肿瘤-）， P40（-）， CK7（+）， TTF-1（+）， Napsin A（+）， Syn（-）， CgA（-）， CD56（-）， CK8/18（+）， P53（散在弱+） ，癌胚抗原（carcinoembryonic antigen, CEA）（+）， Ki-67（+）， CK20（+）， CR（散在间皮+，肿瘤-），MC（散在间皮+，肿瘤-），Villin（+），MLH1（+），PMS2（+），MSH2（+），MSH6（+）。完善腹盆部增强CT未见明显异常。鉴于患者高龄及一般状况较差，未进一步完善胃肠镜检查，腹盆部影像学未见明确消化系统原发肿瘤证据，结合胸腔积液病理形态学及免疫组化，依据WHO诊断要点最终诊断为PEAC。但因未行胃肠镜检查，仍存在隐匿性消化道原发灶不能完全排除的局限。2025年4月9日改用第三代EGFR-TKIs阿美替尼，每次110 mg，每日1次。治疗仅1周后（2025年4月15日）复查影像学提示胸腔积液较前明显增多，疗效评估为疾病进展（图1C、1D）。考虑患者高龄且因疾病快速进展导致一般状况明显下降，美国东部肿瘤协作组体能状态（Eastern Cooperative Oncology Group performance status, ECOG PS）评分为3分，难以耐受含紫杉醇、铂类等传统静脉化疗方案。2025年4月17日起行芦康沙妥珠单抗治疗，每次200 mg，每2周1次。2025年5月22日复查胸部增强CT仍示右肺中下叶实变影较前增大，病灶较前进展（图1E、1F）。患者一般情况持续恶化，2025年6月10日予特瑞普利单抗240 mg联合贝伐珠单抗300 mg治疗，共2个周期，患者喘憋症状仍持续加重。后家属放弃进一步诊疗，患者于1个月后因呼吸、心力衰竭死亡，总生存期为9个月。

**图 1:**
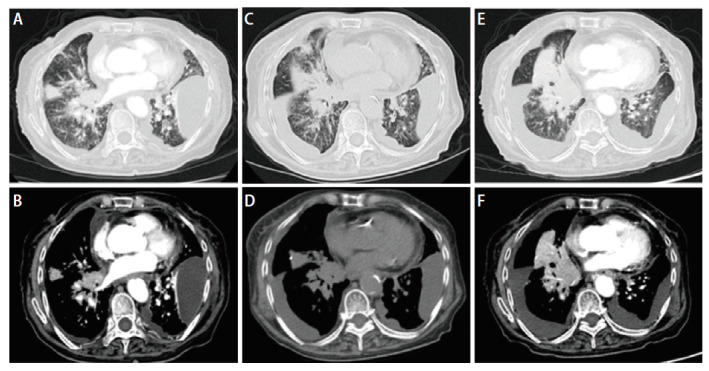
患者的胸部CT影像。A、B：胸部增强CT（2025年4月7日）（A：纵隔窗，B：肺窗）示：左肺上叶肿物、左肺上叶及右肺下叶实变影；双侧胸腔积液；C、D：胸部平扫CT（2025年4月15日）（C：纵隔窗，D：肺窗）示：左肺上叶肿物，左肺上叶及右肺下叶实变影较前无著变，右侧胸腔积液较前增多；E、F：胸部增强CT（2025年5月22日）（E：纵隔窗，F：肺窗）示：右肺中下叶实变影较前明显增大。

**图 2:**
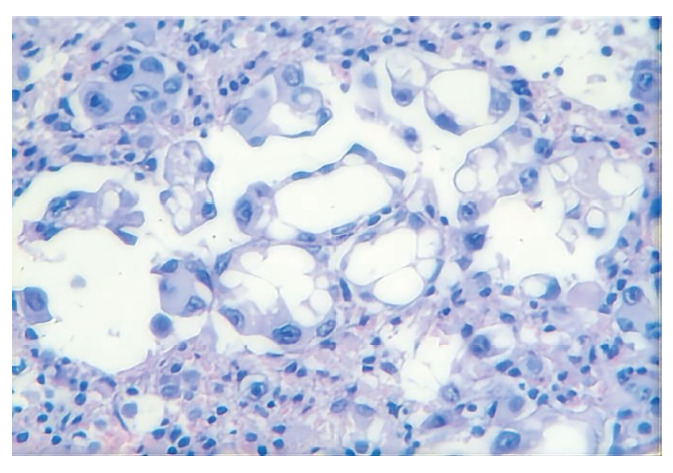
HE染色图片（胸腔积液）示腺癌细胞分布（×400）。肿瘤细胞呈腺管样或微乳头状排列，部分区域可见胞浆内黏液，结合免疫组化符合肺肠型腺癌。

## 2 讨论

PEAC是NSCLC的一种特殊亚型，好发于老年女性，其组织形态学及免疫表型与转移性结直肠腺癌高度相似，给临床诊断带来较大挑战。Zhao等^[5]^研究指出PEAC患者通常无特异性临床表现，常以咳嗽、咯血、发热、胸痛等非特异性呼吸系统症状起病。影像学方面，PEAC与常规侵袭性肺腺癌患者在分叶征、毛刺征、胸膜凹陷征和胸腔积液等表现方面无显著差异。因此，PEAC临床表现和影像学特征难以作为确诊依据，仍需依赖病理形态学特征及免疫组化标志物来进行综合判断。

PEAC的细胞结构主要（>50%）类似于结直肠腺癌，肿瘤细胞常呈高柱状，胞浆嗜酸性，具刷状边缘，细胞核较大且核仁明显。组织学上，PEAC通常至少表达一种肠道分化相关标志物，如CDX2、CK20、MUC2或Villin，而肺部来源标志物如CK7、TTF-1和Napsin A等可能呈部分阳性或完全阴性^[6]^。Nottegar等^[7]^对46例 PEAC患者的病理特征分析发现，CK7和CDX2的阳性率为100%，而TTF-1、CK20和MUC2的阳性率分别为45.6%、32.6%和32.6%，提示CK7联合CDX2可作为PEAC的重要免疫组化标志物。本例患者以上肢麻木、疼痛等骨转移相关临床症状为首发表现，支气管镜活检初步诊断为肺腺癌。进一步免疫组化复核发现，肿瘤组织同时表达结直肠癌标志物（CK20、Villin）和肺癌标志物（CK7、TTF-1）。结合影像学检查和消化系统评估，基本排除胃肠道原发灶后，最终明确诊断为PEAC。

目前，PEAC的治疗多参照NSCLC诊疗策略。对于绝大多数（>90%）早期PEAC患者，手术切除是首选治疗方式。对于无法手术的晚期PEAC患者，最优全身治疗尚存在争议。化疗是PEAC的主要辅助治疗手段，最常见的治疗方案是卡铂联合紫杉醇。然而，既往关于化疗在PEAC中的研究多为小样本回顾性报道，且临床IV期患者化疗后的生存获益差异较大（2-12个月不等），提示疗效可能受到患者PS评分、肿瘤负荷及合并症等因素的显著影响^[4]^。鉴于PEAC在形态学及免疫表型上与结直肠腺癌存在一定相似性，部分研究者尝试沿用结直肠癌化疗方案，如奥沙利铂或伊立替康联合氟尿嘧啶等，但总体疗效并不理想^[1]^。近年来，PEAC精准治疗的探索也在逐渐深入。Nottegar等^[7]^在46例PEAC患者的研究中发现，*KRAS*第12密码子突变率高达60.9%，是最常见的突变基因，而表皮生长因子受体（epidermal growth factor receptor, *EGFR*）、神经母细胞瘤RAS病毒癌基因同源物（neuroblastoma RAS viral oncogene homolog, *NRAS*）、*B-Raf*原癌基因丝氨酸/苏氨酸蛋白激酶（*B-Raf* proto-oncogene, serine/threonine kinase, *BRAF*）突变、转染重排原癌基因（rearranged during transfection proto-oncogene, *RET*）融合和*EML4-ALK*重排等相对少见。目前关于PEAC接受EGFR-TKIs治疗的有效性仍缺乏一致结论。本例患者为*EGFR*外显子19缺失突变伴PD-L1高表达（TPS=80%, CPS=82）的PEAC，接受多代EGFR-TKIs治疗疗效欠佳，表现出对EGFR-TKIs原发耐药，同时多次基因检测均未检出*EGFR* T790M、*KRAS*、*TP53*、磷脂酰肌醇-4,5-二磷酸3-激酶催化亚基 α（phosphatidylinositol-4,5-bisphosphate 3-kinase catalytic subunit alpha, *PIK3CA*）或间质上皮转化因子（mesenchymal-epithelial transition factor, *MET*） 扩增等已知耐药相关共突变。既往研究^[8]^认为这种原发耐药可能由治疗前已存在的旁路激活、拷贝数异常或融合等基因组改变，或与药物耐受持续（drug-tolerant persister, DTP）相关的表观遗传与肿瘤微环境重塑共同驱动。因此，尽管本例多次检测未提示明确耐药位点或典型旁路通路异常，但现有检测结果仍可能受限于肿瘤异质性、取样部位差异等相关因素，难以完全排除结构变异或拷贝数改变等“隐匿耐药”机制的存在。

此外，队列与荟萃分析^[9]^提示，在*EGFR*基因突变NSCLC人群中，PD-L1高表达与EGFR-TKIs疗效不佳、早期进展及*EGFR *T790M突变率较低有关，提示其可能是原发耐药的风险标志，这与我们病例中先后接受第一代至第三代EGFR-TKIs治疗仍快速进展的临床表现相符。研究^[10]^表明，*EGFR*突变型肺腺癌更倾向于表现出非炎症表型，肿瘤浸润性免疫细胞较少，这提示PD-L1多为驱动通路相关上调而非“适应性”表达。因此，*EGFR*突变型肺腺癌中PD-L1高表达更可能源于除EGFR以外致癌信号的激活或存在更深层次的基因组不稳定与肿瘤微环境重塑，从而驱动EGFR非依赖型耐药^[11]^，这也可能是本病例未从后续ICIs联合贝伐珠单抗治疗中获益的原因之一。

另一方面，PEAC是一种混合型腺癌，其肿瘤内部肠型与非肠型分化成分之间可能存在明显的分子异质性。Yang等^[12]^报道1例*EGFR*突变型PEAC伴T790M及微卫星高度不稳定性（microsatellite instability-high, MSI-H）的患者，在接受一线埃克替尼治疗后进展，对奥希替尼及免疫治疗亦无明确获益。*EGFR*突变可能主要存在于普通肺腺癌成分，而肠型成分对EGFR-TKIs不敏感，在治疗过程中逐渐成为优势克隆，进而导致原发耐药。因此，需进一步加强对PEAC发病机制及肿瘤内异质性的分子水平研究，以指导个性化治疗策略。

TROP2是一种35 kDa的跨膜糖蛋白，属于上皮细胞黏附分子（epithelial cell adhesion molecule, EpCAM）家族，在多种上皮癌中过表达，并且通常与肺腺癌预后不良相关。Baldacci等^[13]^研究表明TROP2是*EGFR*基因突变NSCLC经EGFR-TKIs治疗后DTP细胞中的首要靶点。因此，在EGFR-TKIs短时间耐药且患者高龄、一般状态较差及肿瘤负荷较大的背景下，我们选择TROP2-ADC作为二线治疗。然而，该患者在接受TROP2-ADC治疗后病情仍持续快速进展。我们认为TROP2-ADC疗效不佳可能与药物暴露不足、肿瘤内异质性及内吞与胞内转运差异等多因素有关，未来仍需在PEAC患者中开展更系统的前瞻性研究，以验证TROP2-ADC的临床获益。

综上，PEAC是一种具有独特组织学及分子学特征的肺腺癌的特殊亚型，异质性显著。未来研究应重点关注PEAC的多组学特征解析，包括驱动基因通路交互、免疫微环境特征以及肠型与非肠型成分间的克隆演化规律。同时，TROP2-ADC、ICIs等治疗策略在该亚型中的作用仍需进一步探讨，而针对PEAC的精准分型与个体化治疗可能将成为改善预后的关键方向。本例的局限性在于患者高龄及一般情况限制，未能完成胃肠镜检查，尽管腹盆部增强CT未见明确消化系统原发肿瘤证据，仍不能绝对排除隐匿性消化道原发灶的可能。后续类似病例建议在条件允许时完善胃肠镜及必要的消化系统评估，以进一步提高PEAC诊断的严谨性。
